# Experimental study on the influence of virtual tourism spatial situation on the tourists’ temperature comfort in the context of metaverse

**DOI:** 10.3389/fpsyg.2022.1062876

**Published:** 2023-01-04

**Authors:** Xiao-Ting Huang, Jiahui Wang, Zhihui Wang, Linqiang Wang, Chenfei Cheng

**Affiliations:** ^1^Department of Culture and Tourism Management, School of Management, Shandong University, Jinan, Shandong, China; ^2^Isenberg School of Management, University of Massachusetts, Amherst, MA, United States

**Keywords:** virtual tourism, tourism spatial situation, thermal sensation, temperature comfort, experimental research

## Abstract

The Metaverse is a new application of the internet and social form which integrates a myriad of new technologies. It can not only create a parallel space that is closely connected to the real world while highly independent, but also bring the immersive experience of virtual scenarios without delay. The virtual tourism space situations that integrate realistic visual, audio, and temperature sensations can restore the real tourism environment to the greatest extent, and improve tourists’ perception and satisfaction with the experience. The purpose of this experimental lab study is to examine the effects of the virtual tourism audio-visual conditions and environmental temperature on tourists’ thermal sensation and temperature comfort. VR equipment and microclimate simulation technology was applied in a 3 × 2 × 2 experimental design (*n* = 180), simulating the virtual tourism scenarios. Electrocardiogram devices were also employed to assess participants’ physiological indicators. Study results suggest that: (1) Virtual tourism spatial situations (environmental temperature and audio-visual conditions) significantly affect participants’ thermal sensation and part of the physiological indicators; (2) Virtual tourism spatial situations (environmental temperature and audio-visual conditions) significantly affect participants’ temperature comfort; and (3) Physiological responses (indicators) mediate the effect from tourism spatial situations to temperature comfort. The study mainly contributes to the literature about virtual tourism experience and spatial situations under the concept of the Metaverse, as well as provides theoretical and managerial implications for the development of “immersive” virtual tourism scenarios.

## 1. Introduction

The term ‘metaverse’ first appeared in a science fiction novel “Avalanche,” which depicts a vast virtual reality world parallel to the real world, where people who are geographically isolated from each other can communicate through their respective “avatars” for entertainment (Dionisio et al., 2013). The metaverse itself is not a technology, but an idea and concept. As a comprehensive integrated application of multiple digital technologies, it needs to integrate different new technologies, such as 5G, artificial intelligence, big data, etc. (Bourlakis et al., 2009; Choi and Kim, 2016), building a fully virtualized world. In general, the Metaverse will surpass previous experiences in terms of “virtuality,” “immersion,” and “connectivity” (Collins, 2008; Barassi and Treré, 2012), redefining and changing the existing consumption habits of consumers. The Metaverse is gaining momentum, and “immersion” is one of the core selling points of the metaverse, which has been applied in virtual tourism (Schwarz et al., 2012; Burton and Schlieman, 2021). In the field of virtual tourism, Disney has taken the lead in interpreting its metaverse strategy as follows, that is, using technologies such as artificial intelligence, virtual reality, and robotic Internet to improve the immersion and personalization level of the park (Mine et al., 2012).

In the metaverse, how can virtual tourism make the tourists feel the most immersive experience? How can virtual tourists feel the temperature changes and the seasons change during their travel? All these are inseparable from the construction of virtual tourism space (Zheng et al., 2010). A diversified and immersive virtual tourism experience is an important part of the metaverse (Singh et al., 2020). The information exchange between virtual tourists and the surroundings happens all the time (Trunfio and Campana, 2020). Different virtual tourism spatial situations bring different virtual tourism experiences to the tourists (Loureiro et al., 2020), a simulated virtual tourism space scenario based on the integration of sight, hearing, and temperature sensations will bring tourists an unprecedented immersive tourism experience (Bogicevic et al., 2019). Through a multi-sensory systems (visual, hearing, touch, etc.) and previous knowledge, memory, and experience in the real world, the experiencer acquired information from the virtual tourism space and form the temperature comfort perception, such as cold, heat, comfort, or discomfort (Jendritzky et al., 2012; Hemmatjo et al., 2017).

Virtual tourists’ temperature comfort perception is an important part of shaping the “authenticity and immersion” of virtual tourism space (Riva et al., 2011). Environmental temperature can affect the experiencer’s temperature comfort (Lam et al., 2018), but considering the function of the multi-sensory system and previous knowledge, memory, and experience, virtual tourists under different tourism spatial situations may have different comfort feeling (Mieczkowski, 2010). Although there are existing studies have explored the influence of spatial context on human thermal sensation and physiological indicators through the experimental method (Ma et al., 2019), some scholars have used the microclimate laboratory to simulate the tourism space situation (Fabbri et al., 2020), the virtual tourism space situation has not yet to be discussed. The existing studies only consider the influence of a single sensory on temperature comfort (Schott and Marshall, 2018). the research on the influence of multi-sensory interaction on temperature comfort is lacking. Therefore, it is necessary to deeply discuss the temperature comfort of virtual tourists under the interaction of visual and auditory multisensory.

Experimental research on virtual tourism has received increasing attention, investigating the specific sensory experiences of virtual tourists by controlling for variables or situational settings (Huang et al., 2020). Photos, videos, virtual reality equipment, and other instruments were often used in the experiment, and physiological indicators were monitored in real time through medical equipment such as heart rate monitors and EEG monitors (Dieck et al., 2018; Marasco et al., 2018). Different from previous virtual tourism experimental research, this experiment will simulate the multi-sensory scene of the future metaverse to the greatest extent possible, to deeply explore the effect of virtual tourism experience. This study presented the virtual tourism space situation scene through VR technology, and the real physical environment is simulated by controlling the environmental temperature, wind speed, humidity, and illuminance through the tourism simulation micro-climate cabin. At the same time, participants’ physiological indicators, such as blood oxygen, pulse rate, blood pressure, heart rate, and respiratory rate were monitored by ECG, and questionnaires about thermal sensation and temperature comfort were asked to fill out. This study is devoted to using an experimental method to analyze the influence of multi-sensory composite factors on the virtual tourists’ temperature comfort, thereby contributing to the sustainable development of the virtual tourism industry from the perspective of the Metaverse.

## 2. Research background and hypothesis development

### 2.1. Metaverse research

The metaverse was just a scientific or fiction concept before 2021 (Girvan, 2018), Neal Stephenson described the metaverse as follows: “Put on headphones and eyepieces, establishing the connection with a terminal, and then you can enter a virtual space that is simulated by computer, which is parallel to the real world” (Perry and Williams, 1995). In March 2021, the online game company Roblowas x was successfully listed on the New York Stock Exchange with the concept of “metaverse,” and it is the first time that the “metaverse” developed from fiction to the stage of practical application (Joo-Eon, 2021). With the development of media and information technology (Williams and Hobson, 1995), people can employ smart devices and the Internet effectively, breaking through the barriers between virtual and reality, and changing people’s lifestyles and cognition (Mueller, 1998; Martinez-Grana et al., 2013). The current development of the Internet is in the stage of Web 2.0 (Razmerita et al., 2009), as the Internet is still developing rapidly, both the academic researchers and the industry both are discussing the future of the Internet. Web3.0 was proposed based on Web2.0 (Hendler, 2009), the Web 3.0 stage is a highly people-centric network, which helps realize the “interconnection of all things” (Aghaei, 2012), making the metaverse change from the “possible world” in literature to one the “virtual tourism” that people can see and touch (Bonnard et al., 2019).

The metaverse is essentially virtualization and digitization of the real world (Russell, 2017). In the metaverse era, to realize the six types of human sensation (sight, hearing, smell, taste, touch, and consciousness), it is necessary to integrate different technologies (Matsubara and Oguchi, 2010; Fei et al., 2019), such as artificial intelligence, digital twins, blockchain, cloud computing, robotics, brain-computer interface, 5G, 6G, etc. These technologies are essentially important to create an ideal virtual world through the perfect connection between virtual and reality (Ariyadewa et al., 2010; Suzuki et al., 2020). The existing research on the metaverse mainly focuses on technical support, application scenarios, and development models (Ben et al., 2018; Gao et al., 2022), ignoring the users’ experience of the metaverse. Considering the booming virtual tourism industry, it is significant to explore how to provide tourists with the most immersive virtual tourism experience.

### 2.2. Research on virtual tourism

In the mid-1980s, the term “virtual tourism” was first proposed to describe human-machine interactions, emphasizing that “virtual reality has the technical potential to recreate situations for participants” (Steuer, 1992; Zyda, 2005). Perry Hobson and Williams first introduced virtual reality into tourism in 1995, they pointed out that “virtual tourism” is a new business form created by the combination of virtual reality technology and tourism. It can dynamically present the real or non-existent touristic landscape to tourists through the Internet or virtual technology (Bowman and Mcmahan, 2007; Saposnik et al., 2010). With the continuous development of technologies such as GIS, 3D visualization, and virtual reality (Brown and Green, 2016), virtual tourism allows tourists to have an immersive travel experience without leaving home (Lin et al., 2020).

The COVID-19 pandemic had a huge impact on the tourism industry, which brought and real-world tourism has come to a standstill (Gssling et al., 2020). Virtual tourism is a good alternative when tourists cannot visit a real touristic destination (Lee et al., 2020), and plays the dual role of a temporary product during the crisis and a promotional tool after the crisis (El-Said and Aziz, 2021). In addition, the development of information technology has improved the quality of virtual tourism experience (González-Rodríguez et al., 2020), attracting more virtual tourists (Sigala, 2020). Virtual tourism provides a convenient choice for tourists to visit protected historical sites or hard-to-reach places (such as space) through the way of immersion, imagination, and interaction (Nolin et al., 2016; Wagler and Hanus, 2018), especially creating barrier-free tourism activities for the disabled, the elderly, and other restricted people (Cho et al., 2002), which break through time and space constraints and economic shackles to a certain extent (Ritchie et al., 2011; Bonetti et al., 2018; Dieck and Jung, 2018).

Since the concept of virtual tourism was put forward, the controversy around this topic has never been down. Can virtual tourism replace real tourism? Most scholars firmly believe that virtual tourism can never replace real tourism (Huang et al., 2013; Kim J. et al., 2020). Some scholars also worry that virtual experience will weaken the objective authenticity of the destination (Tan et al., 2014), because the information presented through virtual tourism may be unreliable and distorted (Dueholm and Smed, 2014). However the metaverse promotes the rapid development of virtual technology and enhances users’ ability to obtain high-quality senses (Dinh et al., 1999; Riva et al., 2004), and multi-sensory immersive virtual travel experience becomes possible. The virtual tourism experience must enhance the sense of immersion and the simulation of the scene, and the real environmental temperature can better satisfy the multi-sensory interactive experience of tourists in the tourism space situation.

### 2.3. Research on tourism spatial situation

The concept of “situation” was first proposed by Thomas and Znaniecki in 1919, and “physical-psychological field theory” revealed the relationship between individual behavior and the situation (Borgatti et al., 2009). “A Dictionary of Psychology (3rd ed.)” defined the spatial situation as an environment that affects people’s psychological activities, has specific meanings and symbols, and is composed of various spatial forms (Dade, 2010). Tourism spatial situation refers to the background elements of tourists in the process of tourism activities, including individual elements and spatial environmental elements (Yang et al., 2004; Shi et al., 2005; Deller, 2010). As the background environment of tourism activities, the tourism spatial situation has an important impact on the psychological mechanism of tourists, which mainly affects tourists through multi-sensory interaction (Jansson, 2002).

The existing literature about the relationship between spatial situation and temperature comfort mainly focuses on indoor spatial context, while outdoor spatial context is rarely discussed. In addition, spatial situation research is relatively hot in the field of architecture and landscape design (Vera and Simon, 1993; Richter et al., 2011), and this topic has not received enough attention in the field of tourism. At present, most of the research about the relationship between people’s audio-visual perception and temperature comfort is based on a single sensory factor. There are relatively few studies that consider the effect of multi-sensory interaction (such as audio and visual sensory) on temperature comfort. Therefore, this study attempts to explore the temperature comfort of the tourists under different virtual tourism spatial situations.

### 2.4. Research on tourism temperature comfort

Tourism is a comprehensive activity carried out by tourists in the real environment of a tourist destination, and climate is an important indicator during the process of tourist activities (Grimm and Zilli, 2009). Changes in climate conditions such as temperature and humidity will comprehensively affect the degree of human comfort (Frontczak and Wargocki, 2011), and then have physiological and psychological impacts on the human body (Tsutsumi et al., 2007). Therefore, climate comfort is crucial to tourism activities.

The research on human climatic comfort can be traced back to the 1920s. Scholars such as Houghton proposed the concept of “Physiologically Equivalent Temperature (PET)” in 1923, and become the first person to use empirical models to evaluate climatic comfort (Houghten and Yaglou, 1923). Tourism climate is an important tourism resource and tourist attraction (Brager and de Dear, 1998), and the ideal tourism climate should provide tourists with a sense of comfort. Climate comfort has gradually become an important factor in tourists’ travel decision-making process (Scott and Lemieux, 2010).

Temperature comfort is an important part of current climate comfort research, and research on temperature comfort plays an important role in helping tourists select travel destinations and make travel decisions (Perch-Nielsen et al., 2010). Tourism temperature comfort not only affects the length of the tourism comfort period and the choice of tourist destinations but also affects the tourism activities ways and the function of tourism resources (Abed and Matzarakis, 2018; Zhong et al., 2019). Previous studies have found that temperature comfort is not only influenced by objective environmental factors, but the psychological adaption is also an important factor (such as knowledge, experience, and memory) is also an important factor in tourists’ temperature comfort (Lu et al., 2016). Thus, this study intergrates microclimate and tourists’ temperature comfort into a comprehensive model, and used the experimental method to explore whether and how different virtual tourism spatial situations affect the experiencers’ thermal sensation, physiological indicators, and temperature comfort.

### 2.5. Virtual tourism spatial situation and tourists’ thermal sensation

Thermal sensation refers to the human body’s objective perception of ambient temperature, and temperature comfort is formed under the combined influence of individuals’ physiology and psychology (Gagge et al., 1969). The term human thermal sensation is more objective (Nikolopoulou and Steemers, 2003). It was assumed that environmental temperature would affect human body’s thermal sensation. As the temperature increases, the thermal sensation of the human body will also increase (Yang et al., 2016). Some researchers have discussed the effect of spatial context on thermal sensation, but the spatial situation mainly includes color, layout, environmental temperature, sound, etc. (Zhang et al., 2004; Ali-Toudert and Mayer, 2006; Xu et al., 2021). On the one hand, environmental psychology believes that visual conditions (e.g., color) would affect people’s thermal sensations (Tetsuo et al., 1998; Wang et al., 2018). On the other hand, environmental temperature is an important factor affecting human thermal sensation (Ji et al., 2020). It has been found that environmental sound has a direct impact on human thermal sensation from both subjective and objective perspectives (Yang and Moon, 2019). Noise can increase an individual’s thermal sensation in a specific environment, and pleasant sounds can reduce an individual’s thermal sensation in a specific environment (Qu, 2010; Kilchenmann and Senn, 2015). According to the embodied theory of tourism experience, tourism activities are carried out in different spatial situations, and virtual tourism activities cannot exist without a specific spatial situation. Therefore, the study hypothesis that:

*H1*: Environmental temperature has a significant positive impact on virtual tourists’ thermal sensation.

*H2*: Audio spatial situation has a significant positive impact on virtual tourists’ thermal sensation.

*H3*: Visual spatial situation has a significant negative impact on virtual tourists’ thermal sensation.

### 2.6. Virtual tourism spatial situation and tourists’ physiological indicators

The environmental stress theory indicated that external environmental stimuli can evoke the psychological and physiological responses in the human body. The human body has a thermoregulatory mechanism that is closely related to thermal sensation and temperature comfort. According to the heat balance theory, when the heat storage in the human body is equal to 0, it will be in a comfortable temperature state. As a warm-blooded animal, the human can maintain a relatively constant body temperature despite changes in ambient temperature. Scholars have used skin temperature, heart rate, respiratory rate, and blood pressure as potential indicators to reflect human health and thermal comfort (Xiong et al., 2016; Tussyadiah et al., 2018). Previous studies have shown that temperature and ambient color can significantly affect human heart rate and blood pressure, while human thermal sensation has a positive correlation with average skin temperature, heart rate, and other indicators, and skin temperature is also affected by ambient temperature and sound (Takakura et al., 2015). In addition, temperature and sound also affect the human heart rate. People intend to have a higher heart rate when they are in an integrative environment combined with both higher temperature and noise (Ren et al., 2011). Additionally, individuals’ visual sense directly affects their physiological factors (Sun and Lian, 2016). For example, bright space can reduce heart rate and blood pressure (Peng et al., 2022). Therefore, the study proposed that environmental temperature, hearing sensory, and visual senses would affect the human body’s physiological indicators in virtual tourism.

*H4*: Environmental temperature has a significant impact on virtual tourists’ physiological indicators, and different environmental temperatures have different effects on the physiological indicators.

*H5*: Audio spatial situation has a significant impact on virtual tourists’ physiological indicators, and different audio spatial situations have different effects on the physiological indicators.

*H6*: Visual spatial situation has a significant impact on virtual tourists’ physiological indicators, and different visual spatial situations have different effects on the physiological indicators.

### 2.7. Virtual tourism spatial situation and temperature comfort

The embodiment theory of tourism experience suggests that tourists’ experience is the result of a series of related factors such as perception, body sensory, and environment in the process of tourism (Soica, 2016). Tourists’ travel experience is affected by the “situation” they are in. As part of the travel experience, the temperature comfort is also affected by the tourism spatial situation (Perkins and Debbage, 2016). Temperature comfort is an individual’s subjective satisfactory evaluation of the extent of hot and cold in the surrounding environment, which has been proved significant impact on individual’s perceived temperature comfort (Yun et al., 2012). A large number of studies have also shown that visual and auditory stimuli can affect the human body’s temperature comfort. For example, visual color (Chinazzo et al., 2020a) and environmental spatial layout (Cheong et al., 2007) can affect the comfort of the human body. Sound can have an impact on human thermal comfort (Guan et al., 2020). The noisy environment would influence the temperature comfort of human body (Oquendo-Di et al., 2022), while the beautiful birds singing can calm people down and improve the temperature comfort rating (Yang and Kang, 2005). Some scholars have found that environmental temperature can significantly affect human thermal comfort (Arens et al., 2006; Chinazzo et al., 2018). Thus, the study proposed that environmental temperature and visual and auditory stimuli can affect the temperature comfort of the human body in the virtual tourism context. We hypothesize that:

*H7*: Environmental temperature has a significant positive impact on virtual tourists’ temperature comfort.

*H8*: Audio spatial situation has a significant positive impact on virtual tourists’ temperature comfort.

*H9*: Visual spatial situation has a significant negative impact on virtual tourists’ temperature comfort.

### 2.8. Tourism spatial situations, thermal sensation, physiological indicators, and temperature comfort

**Figure 1:**
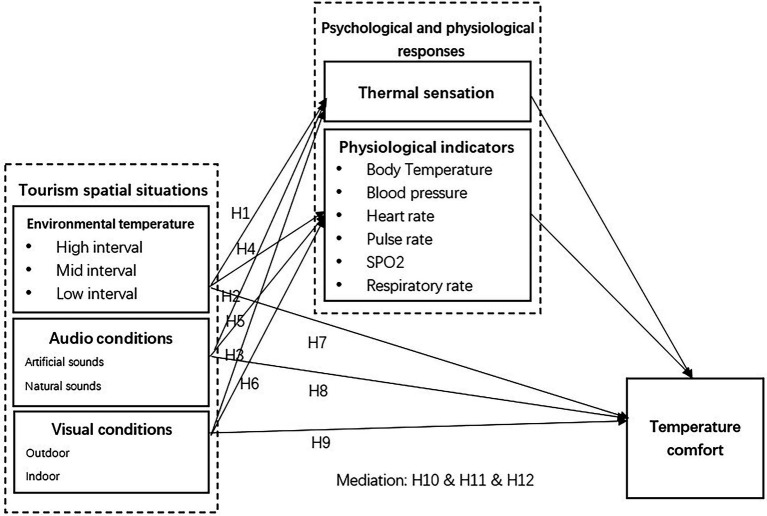
Theoretical model.

In the process of virtual tourism experience, tourists interact with their space through (hearing and vision) information in different virtual tourism spaces, and the two senses interact to form individual thermal sensation (Wang and Zacharias, 2020). Even though tourists stay at the same temperature interval, how they feel the temperature comfort might be different under different tourism space situations based on their own multi-sensory system (Zhang and Zhao, 2008). The spatial context in which tourism activities are located is specific, and temperature comfort is the final judgment formed by combining the individual subjective feelings and physiological indicators perceived by tourists within the spatial context (Gao and Li, 2017). According to the environmental stress theory, external environmental stimuli can lead to individuals’ physiological and psychological stress responses, thus we assume that the tourism spatial situations would have an impact on tourists’ physiological and psychological responses, and both thermal sensation and physiological indicators would influence temperature comfort (Figure 1). Thus we proposed that:

*H10*: Thermal sensation and physiological indicators mediate the effects of environmental temperature on virtual tourists’ temperature comfort.

*H11*: Thermal sensation and physiological indicators mediate the effects of audio conditions on virtual tourists’ temperature comfort.

*H12*: Thermal sensation and physiological indicators mediate the effects of visual conditions on virtual tourists’ temperature comfort.

## 3. Methodology

### 3.1. Research facilities

**Figure 2:**
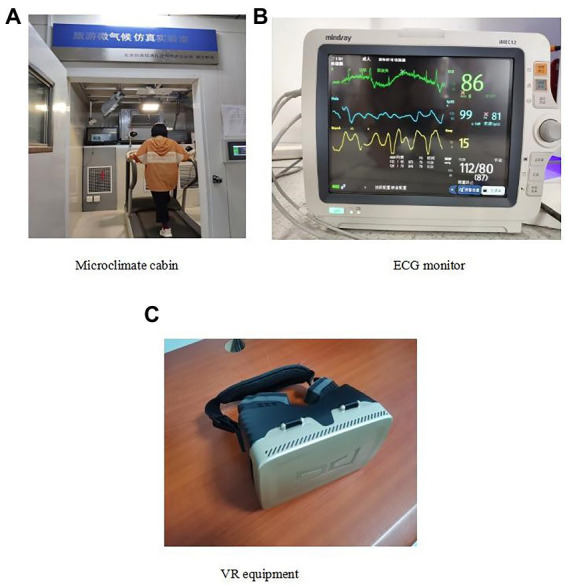
Experimental facilities (**A**: Microclimate cabin; **B**: ECG monitor; **C**: VR equipment). Images reproduced with the permission of Zhang Linlin.

A mixed design laboratory experiment was conducted in a tourism microclimate simulation laboratory at a university in eastern China, from March 2021 to March 2022. The laboratory is equipped with a tourism simulation microclimate cabin (Figure 2A); a treadmill (to simulate the activity state of tourists); an ECG monitor (to measure the physiological data of tourists; Figure 2B); a VR helmet (to simulate an immersive tourism situation; Figure 2C), etc. The size of the tourism simulation micro-climate cabin is 2 m × 2 m × 3 m. There are temperature sensors, air humidifiers, LED lamps, and air vents in the cabin. The indoor temperature, humidity, wind speed, light, and other environmental factors can be monitored through the control panel outside the cabin.

### 3.2. Research design

#### 3.2.1. Environmental temperature intervals

The tourism experience embodiment theory advocates that the daily life tourism research; therefore, the temperature index of virtual tourism space situation in this paper mainly selects the temperature level in the usual environment. Physiologically Equivalent Temperature (PET) has been frequently used in tourism climate studies to estimate participants’ thermal comfort (Walther and Goestchel, 2018). The environmental temperature interval division in this study refers to the PET index, that is, in a certain indoor or outdoor environment, the environmental temperature which can keep the human body temperature and environment temperature at the same thermal state. The “seasonal anchor point method” has been proved to be a rigorous somatosensory grading standard in previous studies (Yu and Li, 2019). Combined with the PET index and seasonal anchor point method, the environmental temperature interval in this study was divided into three types, namely high interval (23.6–30.0°C), mid interval (17.6–23.5°C), and low interval (11.5–17.5°C; Table 1).

**Table 1:** Experiment conditions.

Audio-visual conditio Environmental temperature	a:VR1+ artificial sounds	b:VR1 + natural sounds	c:VR2 + artificial sounds	d: VR2 + natural sounds
High interval A:23.6–30.0	Aa	Ab	Ac	Ad
Mid interval B:17.6–23.5	Ba	Bb	Bc	Bd
Low interval C:11.5–17.5	Ca	Cb	Cc	Dd

#### 3.2.2. Audio-visual conditions of virtual tourism

The tourism spatial situation is relatively complex, and tourists mainly acquire external information through sight and hearing sensations during the process of tourism activities (Liu et al., 2020). Previous studies have indicated that the openness and closure of people’s visual space will affect their perception of perceived physiological temperature (Chinazzo et al., 2020b). This study selects the indoor and outdoor design experiments of scenic spots that are common in the previous tourism visual space research, to explore the influence of virtual tourism visual space characteristics on thermal sensation and temperature comfort. Acoustical ecology believes that artificial sounds and natural sounds showed huge differences due to their different physical properties such as frequency and intensity, which could affect tourists’ experience and perception (Yang and Kang, 2013). At the same time, due to their differences in formal attributes such as volume, pitch, and timbre, tourists would have different thermal sensations and perceive different levels of temperature comfort (Ravicz et al., 2014). The visual scene selection is based on whether there is a top cover design, and two typical virtual tourism visual space situations (VR1 and VR2) were selected. Among them, the VR1 situation is a relatively empty outdoor space; VR2 is a virtual tourism indoor space. At the same time, previous studies have also found that sound can directly affect the temperature comfort of the human body. The experiment used two typical sounds as easily controlled artificial sounds (sounds of tour guides and media broadcasts) and natural sounds (running water and birdsong), to explore the impact on virtual tourists’ thermal comfort. According to the video type and sound type, the experimenters were divided into four groups for control experiments, as shown in Table 1.

### 3.3. Measurements

#### 3.3.1. Survey measurements

The survey questionnaire in this study assessed participants’ thermal sensation and temperature comfort, as well as the socio-demographic information. The thermal sensation was measured by the thermal sensation voting scale (TSV), which is generally recognized and used by researchers (Fanger, 1967). TSV measured participants’ thermal sensation with seven points (1 = cold, 2 = slightly cold, 3 = cool, 4 = neutral, 5 = warm, 6 = slightly hot, and 7 = hot). The temperature comfort was measured by the temperature comfort voting scale (TCV), which was measured through 0–4 points (0 = comfortable, 1 = slightly uncomfortable, 2 = uncomfortable, 3 = very uncomfortable, and 4 = intolerable; Kim M.J. et al., 2020).

#### 3.3.2. Physiological measures

The physiological indicators in the study include body temperature, blood pressure, heart rate, pulse rate, SPO2, and respiratory rate. Except body temperature was assessed by the thermometer, other physiological indicators were monitored by the iMEC 12 devices in real-time. The detailed information about physiological indicators has been shown in Table 2. The physiological indicators during the virtual tourism experience revealed their physiological thermal response to different tourism spatial contexts.

**Table 2:** Experimental instruments.

Instruments	Simulation/ test content	Units
Microclimate warehouse	Air temperature	°C
	Relative humidity	%
	Wind speed	m/s
	Illuminance	Lux
ECG measuring instrument	PR (Pulse rate)	Bmp
	HR (Heart rate)	Bmp
	SpO_2_ (Pulse oxygen saturation)	%
	NIBP (Blood pressure)	mmHg
	RR (Respiratory rate)	rpm

### 3.4. Sampling procedure

#### 3.4.1. Sampling selection

A total of 180 volunteers were recruited by a University’s tourism behavior laboratory (TBL) WeChat public account, including 90 males and 90 females. The participants were mainly aged between 18 and 30 years old, with an average age of 28.47 years; the BMI selection standard was between 20 and 23, with an average value of 21.61. In order to prevent the influence of the thermal resistance of clothes on the experiment results, all participants wore short-sleeved T-shirts and single trousers uniformly. At the same time, all the volunteers should have no history of heart disease and other health problems, have good sleep and diet before the experiment, and do not participate in strenuous exercise the day before the experiment.

During the experiment, according to the combination of three environmental temperature intervals and four types of tourism audio-visual conditions, 12 experimental conditions were formed (Table 1). The participants were randomly divided into three groups, each group containing 60 people. The first group of participants experience four types of virtual tourism audio-visual situations in turn at high-environmental temperature intervals, the second group experience four types of audio-visual situations at the mid-temperature interval, and the third group conducted the same experiment at low environmental temperature. That means each participant experienced four sets of the experiment at a certain environmental temperature interval and participants experienced four types of situation in random. Existing literature has revealed that thermal sensation will reach a relatively stable state when the human body is in an activity for 15 min (Gutierrez-Avellanosa and Bennadji, 2015). Therefore, the duration of each group of experiments is 15 min. After each group of experiments, participants were asked to indicate their thermal sensation and temperature comfort. In order to avoid the impact of the previous experimental process on the subsequent experiments, after each experiment, the participants were get out of the micro-climate cabin, and sit for 10 min to recover to a resting state.

#### 3.4.2. Experiment steps

The specific experimental steps are as follows: First, participants need to sit quietly for 15 min before the experiment and keep their minds calm. During this period, participants were asked to fill in the experimental informed consent form and the participants’ basic information questionnaire, to let participants get a comprehensive understanding of the experimental process and precautions. Secondly, the tourism simulation microclimate cabin was used to establish a manipulated virtual tourism environment by controlling the environmental temperature, humidity, wind speed, treadmill speed, and so on. Thirdly, the participants were asked to dress in an experimental cloth, put on the electrocardiograph and VR equipment, and entering the tourism simulation microclimate cabin. While watching the each VR video, the ECG was recording the physiological responses. After watching every video, participants need to report their feelings by filling in questionnaires. The participants will not start the next experiment until they have rested and are back in a stable condition.

### 3.5. Data analysis

SPSS 23.0 software was used for data analysis. First of all, the homoscedasticity has been checked. The normality assumption was confirmed as skewness and kurtosis values of each variable were within the acceptable threshold (Field, 2009). Next, multivariate ANOVA (MANOVA) was conducted to test the main effects of the independent variable with and dependent variables. Lastly, Model 4 of the PROCESS v3.3 macro was used to test mediation effects in H10, H11, and H12.

## 4. Results

### 4.1. Main effects of environmental temperature of virtual tourism

Multivariate ANOVA was conducted to compare dependent measures among four types of virtual tourism contexts. The independent variable in the MANOVA were environmental temperature conditions (high vs. medium vs. low interval), audio-conditions (artificial sounds vs. natural sounds), and visual conditions (outdoor vs. indoor). The dependent variables in the theoretical framework were thermal sensation, physiological sensation (body temperature, blood pressure, heart rate, pulse rate, SPO2, and respiratory rate), and temperature comfort. The MANOVA results revealed that temperature condition has a significant effect on the participants’ travel experience such as temperature comfort, with a Pillai’s trace value of 0.798, *F* = 67.532, *p* < 0.001.

The results of the MANOVA test are shown in Table 3. All the dependent variables showed significant differences across temperature conditions, higher environmental temperature evoked higher thermal sensation, body temperature, heart rate, pulse rate, and SPO2, while higher environmental temperature intrigued lower blood pressure. Therefore, the findings support H1 and H4. When virtual tourists travel in a low environmental temperature, participants perceived the worst temperature comfort (*M* = 1.29), and mid-environmental temperature elicit the best temperature comfort (*M* = 0.43). This finding supports H7.

**Table 3:** Main effects of environmental temperature conditions.

Variables	Mean (High interval)	Mean (Mid interval)	Mean (Low interval)	F
Thermal sensation	5.03	3.79	2.43	700.78^***^
*Physiological sensation*				
Body temperature	36.38	35.74	35.15	438.69^***^
Blood pressure	87.49	89.23	90.15	5.07^**^
Heart rate	97.14	95.64	89.40	43.95^***^
Pulse rate	94.38	89.64	81.17	83.20^***^
SPO_2_	97.08	96.91	93.31	57.97^***^
Respiratory rate	21.63	22.21	20.48	11.87^***^
Temperature comfort	0.80	0.43	1.29	143.17^***^

^***^p < 0.001; ^**^p < 0.01; ^*^p < 0.05; and ^n.s.^*p* > 0.05.

### 4.2. Main effects of audio conditions of virtual tourism

The MANOVA results showed that audio context type has a significant main effect with a Pillai’s trace value of 0.064, *F* = 6.932, *p* < 0.001. Table 4 showed audio conditions of virtual tourism only have the main effect on participants’ thermal sensation, part of the physiological sensation (SPO2), and temperature comfort. At the same time, no significant differences were identified in body temperature, blood pressure, heart rate, pulse rate, and respiratory rate. Therefore, these findings support H3 and H6, and partially support H4. When traveling under the natural sounds condition of virtual tourism, participants have lower SPO2 (*M* = 95.23) and are expected to perceive cooler thermal sensation and better temperature comfort. This finding partially supports H2 and H5.

**Table 4:** Main effects of audio conditions.

Variables	Mean (artificial sounds)	Mean (natural sounds)	F
Thermal sensation	3.91	3.59	8.75^**^
*Physiological sensation*			
Body temperature	35.76	35.75	0.01 ^n.s.^
Blood pressure	89.59	88.34	3.14 ^n.s.^
Heart rate	94.22	93.90	0.18 ^n.s.^
Pulse rate	88.72	88.08	0.47 ^n.s.^
SPO_2_	96.31	95.23	9.30^**^
Respiratory rate	21.56	21.32	0.60 ^n.s.^
Temperature comfort	0.98	0.69	27.46^***^

^***^p < 0.001; ^**^p < 0.01; ^*^p < 0.05; and ^n.s.^*p* > 0.05.

### 4.3. Main effects of visual conditions of virtual tourism

The MANOVA results showed that visual context type has a significant main effect with a Pillai’s trace value of 0.174, *F* = 21.428, *p* < 0.001. Table 5 shows that visual conditions of virtual tourism only had a main effect on participants’ thermal sensation, part of the physiological sensation (Respiratory rate), and temperature comfort. There is no significant differences were identified in body temperature, blood pressure, heart rate, pulse rate, and SPO2. Therefore, these findings support H3 and H6, and partially support H4. When traveling under the outdoor condition of virtual tourism, participants have a lower respiratory rate (*M* = 21.04) and are expected to perceive cooler thermal sensation and better temperature comfort. This finding partially supports H3 and H6.

**Table 5:** Main effects of visual conditions.

Variables	Mean (outdoor)	Mean (indoor)	F
Thermal sensation	3.98	3.51	19.38^***^
*Physiological sensation*			
Body temperature	35.78	35.73	0.871 ^n.s.^
Blood pressure	89.41	88.52	1.59 ^n.s.^
Heart rate	94.45	93.67	1.06 ^n.s.^
Pulse rate	89.17	87.62	2.73 ^n.s.^
SPO_2_	96.11	95.43	3.61 ^n.s.^
Respiratory rate	21.84	21.04	6.96^**^
			
Temperature comfort	1.11	0.57	109.22^***^

^***^p < 0.001; ^**^p < 0.01; ^*^p < 0.05; and ^n.s.^*p* > 0.05.

In terms of the interaction effect, there was a significant three-way interaction among environmental temperature, audio situation, and the visual situation on thermal sensation (*F* = 3.45, *p* < 0.05), however, the three-way interaction showed a non-significant influence on other dependent variables. Given the results shown above, we further examine the two-way interaction effect on thermal sensation. The results showed that environmental temperature interacted with audio situation to influence thermal sensation (*F* = 39.03, *p* < 0.05). The interaction between audio situation and visual situation also significantly influences thermal sensation (*F* = 61.76, *p* < 0.05). Additionally, environmental temperature interacted with the visual situation to influence thermal sensation (*F* = 87.38, *p* < 0.05).

### 4.4. Mediating effects of thermal sensation and physiological indicators

Model 4 of the PROCESS v3.3 macro was used to examine the theoretical mediating role of perceived thermal sensation and physiological responses between tourism spatial context and temperature comfort. When testing each independent variable, another two independent variables were added as control variables in the Model 4 of PROCESS.

**Figure 3:**
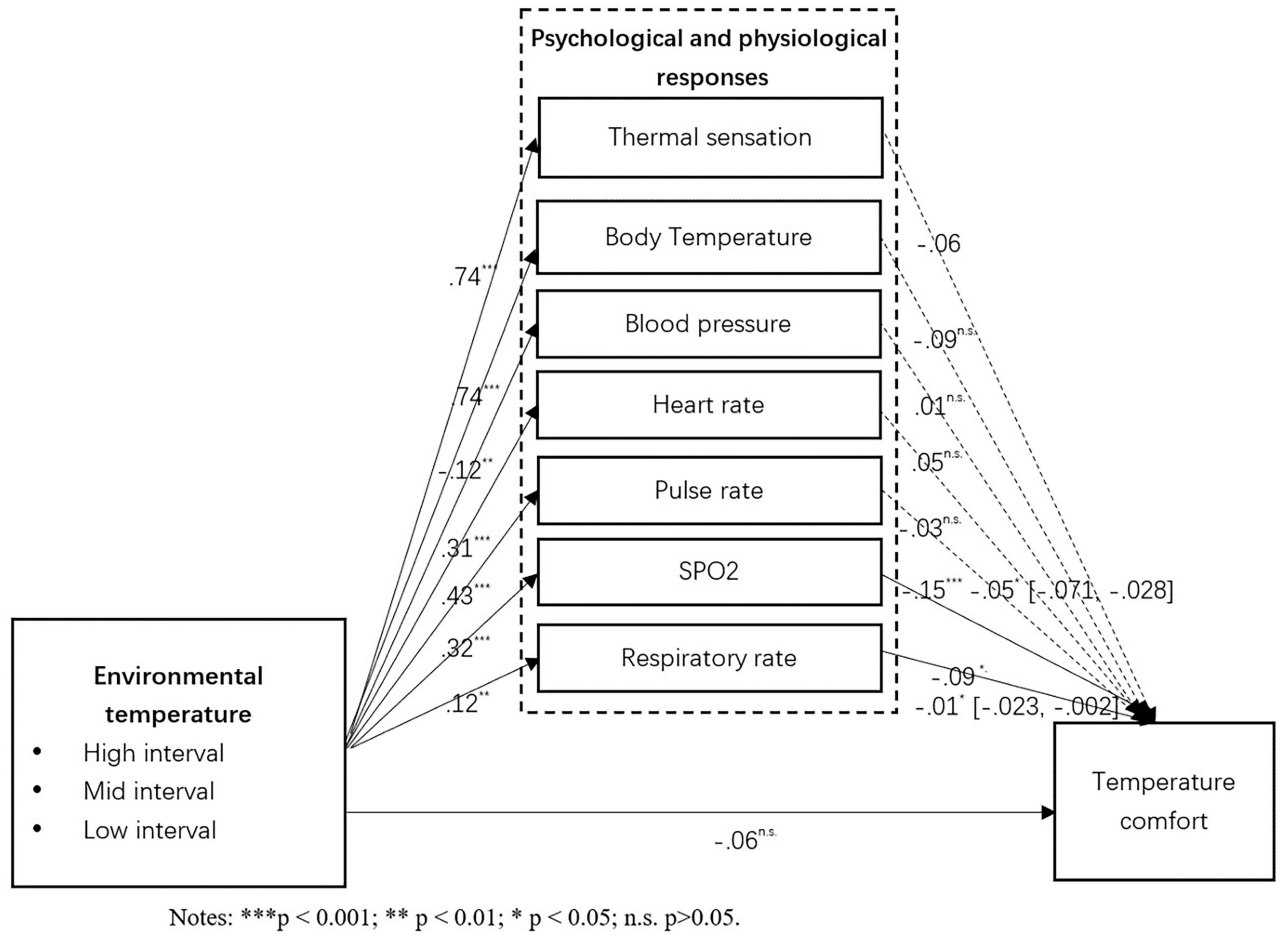
Mediating effects.

**Figure 4:**
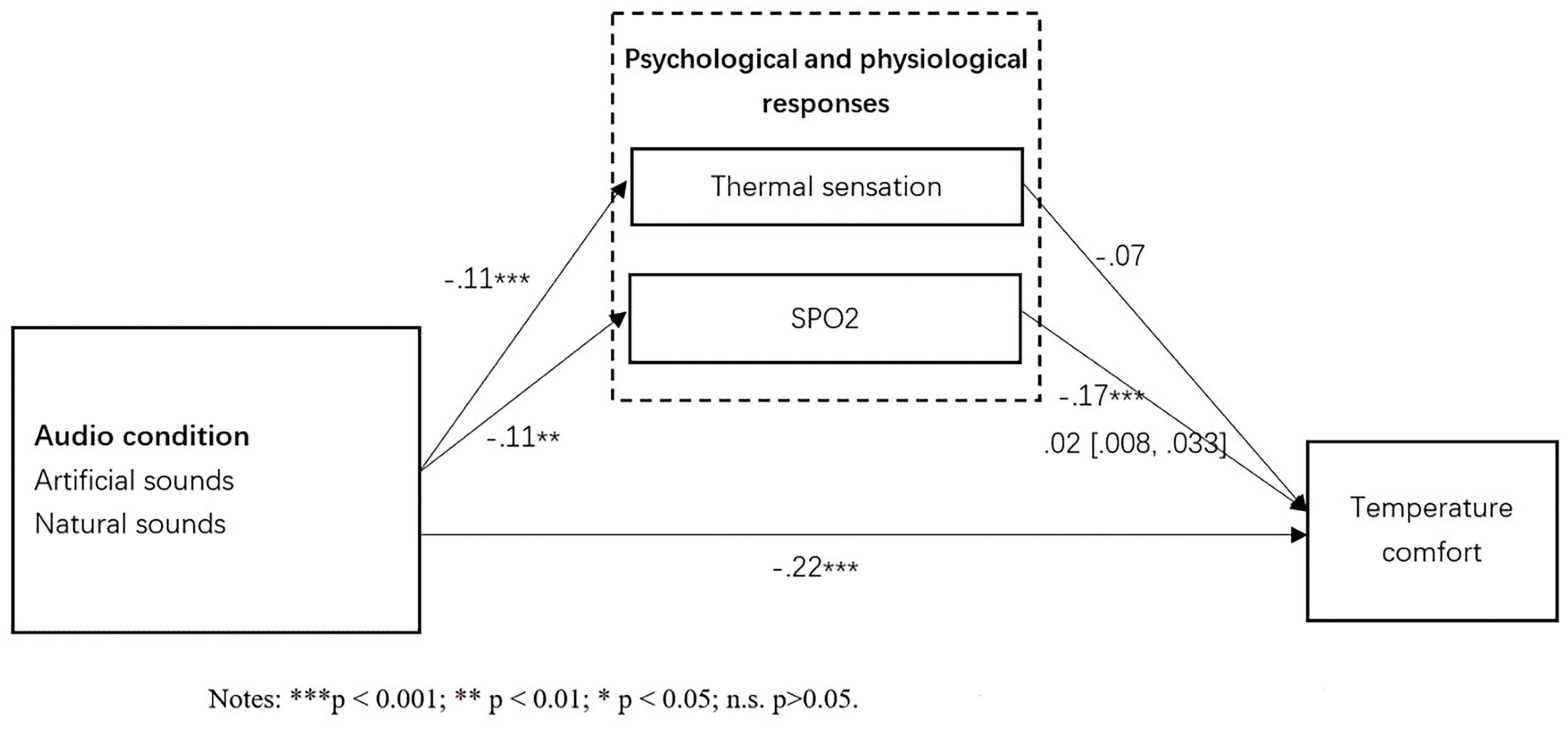
Mediating effects.

**Figure 5:**
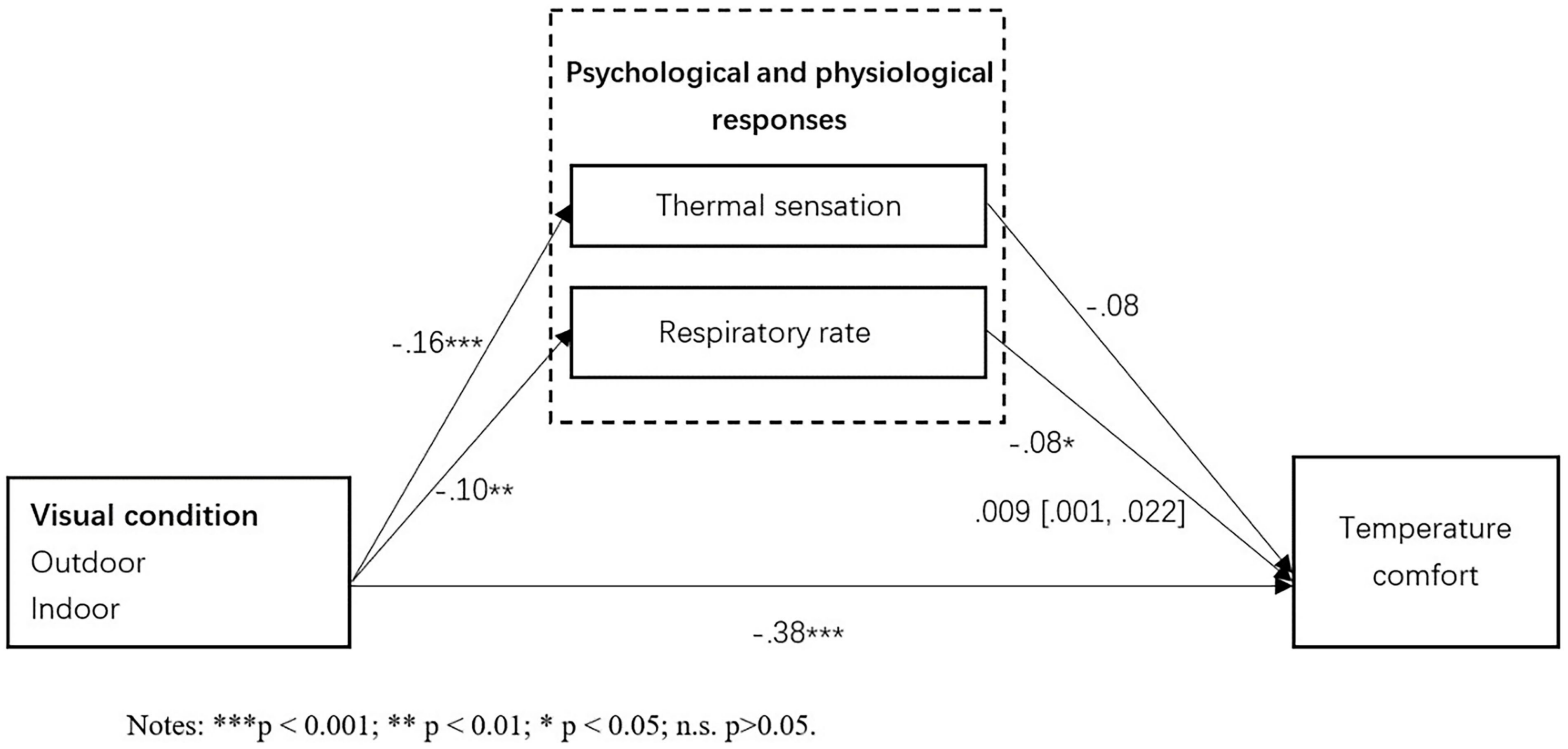
Mediating effects.

As showed in Figure 3, the results showed that a significant mediating effect of the participant’s physiological response SPO2 (indirect effect B = −0.034; 95% CI = [−0.059, −0.011]) and Respiratory rate (indirect effect B = −0.01; 95% CI = [−0.023, −0.002]) between environmental temperature and the corresponding temperature comfort when it is tested with control variables. As shown in Figure 4, the mediating effect of SPO2 (indirect effect B = 0.02; 95% CI = [0.008, 0.033]) has also been confirmed among audio condition and temperature comfort. As shown in Figure 5, the mediating effects of respiratory rate (indirect effect B = 0.009; 95% CI = [0.001, 0.022]) has been confirmed among visual condition and temperature comfort. However, psychological response thermal sensation did not show a significant mediating effect between all three independent variables and temperature comfort. These findings have partially supported H10, H11, and H12.

## 5. Discussion and conclusion

### 5.1. Conclusion

The embodiment theory of tourism experience proposes that the tourism experience is based on specific physical perceptions, which is based on specific physical perception, while virtual tourism in the context of the metaverse is a typical embodied communication, that is, the virtual tourists participate in the tourism process and activities in real. An integrated virtual tourism spatial situation can restore the real tourism scene with the manipulation of realistic sight, hearing, and environmental temperature. At present, the microclimate simulation experiment is a research hotspot of environmental psychology, but few studies have introduced it to the field of virtual tourism, let alone introduced it to the research of the spatial situation of virtual tourism. This study used a microclimate simulation cabin to simulate different types of virtual tourism spatial contexts, and explore their effect on tourists’ thermal sensation, physiological indicators, and temperature comfort, responding to call for the use of VR technology of Wang et al. (2022) to improve the authenticity of the experimental scene.

The current study indicated a significant relationship between the travel spatial situation (environmental temperature and audio-visual spatial context) and thermal responses (thermal sensation and physiological indicators). First of all, this finding is consistent with study of Lu et al. (2016) that air temperature correlated with individuals’ comfort. At the same time, the result is partially different from previous studies, for example, Fanger et al. (1977) found that the ambient sound would not influence subjects’ physiological indicators, while our findings confirmed the relationship between these two variables.

Additionally, this study found a significant relationship between virtual tourism spatial situations and temperature comfort. This deepened study of Tsutsumi et al. (2005) that when the thermal sensation of destination is higher than the thermal sensation at home, people are more likely to feel comfortable.

Moreover, this study has confirmed the mediating role of tourists’ physiological indicators between tourism spatial context and tourists’ temperature comfort. This finding indicated that the tourism spatial situation not only affects tourists’ temperature comfort directly but also affects the temperature comfort indirectly through the function of physiological indicators.

### 5.2. Theoretical implications

The immersive experience of virtual tourism is a manifestation of the metaverse, and the spatial context is particularly important as the key point of the immersive experience. However, previous tourism microclimate simulation experiments were mostly used to discuss the perception of tourists in specific tourism scenarios, and to analyze the behavior of tourists (Wang et al., 2022). This study makes the following theoretical contributions. First, this study enriches the research of virtual tourism from the perspective of tourists’ comfort. Previous research on virtual tourism has mainly discussed the concept and development prospects of virtual tourism theoretically (Guttentag, 2010), or reported the psychology and engagement intention of virtual tourism experiencers (Tavakoli and Mura, 2015). This study explores whether and how different virtual tourism space contexts (audio-visual context and ambient temperature) affect the experiencers’ thermal sensation, physiological indicators, and temperature comfort. Second, this study introduced the microclimate simulation experiment to the virtual tourism spatial situation research. Previous tourism microclimate simulation experiments were mostly used to study the perception of tourists in a specific tourist attraction and analyze the tourists’ behavior (Galagoda et al., 2018). The current study combined the microclimate simulation experiment and tourists’ physiological parameters into the research of virtual tourism spatial situation, enriching our understanding of the effect mechanism of virtual tourism space situation. This experimental study is a considerable innovation because it is different from the traditional “two-dimensional interface and to see three-dimensional effects,” it realized “three-dimensional spatial effects,” which comprehensively improves the realism and immersion of the virtual tourism experience.

### 5.3. Practical implications

This study makes valuable practical contributions to the virtual tourism industry. First, by enhancing the immersive feeling of virtual tourism from the construction of an audio-visual context. The most controversial aspect of virtual tourism is the immersion of experience and the satisfaction of tourists (Cheong, 1995). The findings of the study suggest that the construction of the virtual tourism spatial situation should consider the physiological and psychological data of the experiencer, simulating the tourism situation from the aspects of climate environment, audio, and visual scene, improve the simulation degree of the virtual tourism scene, and enhance the interactivity and immersion of the situation. Second, confirming the developing trend and important role of virtual tourism in the post-COVID-19 period. The “presence” and “immersive experience” emphasized by Metaverse fit the new model pursued by the tourism industry in the digital age, and offer new opportunities for the development of the virtual tourism industry. Third, the study provides theoretical support for the sustainable development of the tourism industry in the metaverse era. The virtual tourism industry is not a comprehensive replacement for the tourism industry, but a supplement and promotion of the tourism industry. Tourist destinations can design and develop targeted virtual tourism activities according to their conditions, to narrow the difference between the low and peak seasons. At the same time, it also provides suggestions for tourists to diversify their travel destination choices, travel strategies, and experience effect previews. The virtual tourism digital scene will connect everything in the future, and continuously integrate and reconstruct the dimensions of business, society, life, and consumption. This research helps to create highly immersive and real-time travel scenes in the three-dimensional space in the future metaverse tourism era.

### 5.4. Limitations and future research

The relationship between different virtual tourism spatial situations and tourists’ thermal sensation, physiological indicators, and temperature comfort is discussed through experimental methods, which is only a preliminary exploration of virtual tourism microclimate research. There are still some limitations:

First, the environmental temperature intervals are not comprehensive enough. The environmental temperature intervals discussed in this study do not involve extremely high temperatures or extremely low temperatures, future studies could explore the tourists’ temperature comfort in the extreme environmental temperature. Second, the experiment sample is not extensive enough due to the epidemic. Future studies could conduct group comparison experiments according to age, physical condition, and region. Additionally, virtual tourism is a comprehensive and complex process, in which various factors affect tourists’ experience and temperature comfort, for this study has shown that example, the video and audio settings in the microclimate cabin might affect the participants’ experimental experience. Future studies could consider other aspects of spatial contexts such as environmental color and population intensity.

## Data availability statement

The raw data supporting the conclusions of this article will be made available by the authors, without undue reservation.

## Author contributions

All authors listed have made a substantial, direct, and intellectual contribution to the work and approved it for publication.

## Funding

This study is supported by grants from the National Natural Science Foundation of China (to XH) (Grant No. 41871138), Shandong University Multidisciplinary Research & Innovation Team of Young Scholars (Grant No. 2020QNQT019) - Jinan City, Shandong Province, China Key Research & Development Plan of Shandong Province - Major Scientific & Technological Innovation Project (Grant No. 2020cxgc010904) - Jinan City, Shandong Province, China.

## Conflict of interest

The authors declare that the research was conducted in the absence of any commercial or financial relationships that could be construed as a potential conflict of interest.

## Publisher’s note

All claims expressed in this article are solely those of the authors and do not necessarily represent those of their affiliated organizations, or those of the publisher, the editors and the reviewers. Any product that may be evaluated in this article, or claim that may be made by its manufacturer, is not guaranteed or endorsed by the publisher.

## References

[ref1] Abed S. S. Matzarakis A. (2018). Quantification of the tourism climate of Algeria based on the climate-tourism-information-scheme. Atmosfera 9:250. doi: 10.3390/atmos9070250

[ref2] Aghaei S. (2012). Evolution of the world wide web: from web 1.0 to web 4.0. Int. J. Web Sem. Technol. 3, 1–10. doi: 10.5121/ijwest.2012.3101

[ref3] Ali-Toudert F. Mayer H. (2006). Numerical study on the effects of aspect ratio and orientation of an urban street canyon on outdoor thermal comfort in hot and dry climate. Build. Environ. 41, 94–108. doi: 10.1016/j.buildenv.2005.01.013

[ref4] Arens E. Hui Z. Huizenga C. (2006). Partial - and whole-body thermal sensation and comfort - part II: non-uniform environmental conditions. J. Therm. Biol. 31, 53–59. doi: 10.1016/j.jtherbio.2005.11.027

[ref5] Ariyadewa P. D. Wathsala W. V. Pradeepan V. Perera R. Atukorale D. (2010). Virtual learning model for metaverses, IEEE, 81-85.

[ref6] Barassi V. Treré E. (2012). Does web 3.0 come after web 2.0? Deconstructing theoretical assumptions through practice. New Media Soc. 14, 1269–1285. doi: 10.1177/1461444812445878

[ref7] Ben F. Shoshana L. Ralph N. (2018). The social metaverse: battle for privacy. IEEE Technol. Soc. Mag. 37, 52–61. doi: 10.1109/MTS.2018.2826060

[ref8] Bogicevic V. Seo S. Kandampully J. A. Liu S. Q. Rudd N. A. (2019). Virtual reality presence as a preamble of tourism experience: the role of mental imagery. Tour. Manag. 74, 55–64. doi: 10.1016/j.tourman.2019.02.009

[ref9] Bonetti F. Warnaby G. Quinn L. (2018). “Augmented reality and virtual reality in physical and online retailing: a review, synthesis and research agenda,” in Augmented Reality and Virtual Reality. 3rd International Conference on Augmented Reality and Virtual Reality (AR and VR). 119–132.

[ref10] Bonnard R. Hascot J. Y. Mognol P. (2019). Data model for additive manufacturing digital thread: state of the art and perspectives. Int. J. Comput. Integr. Manuf. 32, 1–22. doi: 10.1080/0951192X.2019.1690681

[ref11] Borgatti S. P. Mehra A. Brass D. J. Labianca G. (2009). Network analysis in the social sciences. Science 323, 892–895. doi: 10.1126/science.1165821, PMID: 19213908

[ref12] Bourlakis M. Papagiannidis S. Li F. (2009). Retail spatial evolution: paving the way from traditional to metaverse retailing. Electron. Commer. Res. 9, 135–148. doi: 10.1007/s10660-009-9030-8

[ref13] Bowman D. A. Mcmahan R. P. (2007). Virtual reality: how much immersion is enough? Computer 40, 36–43. doi: 10.1109/MC.2007.257, PMID: 36405877

[ref14] Brager G. S. de Dear R. J. (1998). Thermal adaptation in the built environment: a literature review. Energ. Buildings 27, 83–96. doi: 10.1016/S0378-7788(97)00053-4, PMID: 35915820

[ref15] Brown A. Green T. (2016). Virtual reality: low-cost tools and resources for the classroom. TechTrends 60, 517–519. doi: 10.1007/s11528-016-0102-z

[ref16] Burton N. Schlieman T. (2021). User response to extended reality sponsorship activations on social media: exploring impressions of gopro's use of 360° video in marketing. J. Interact. Advert. 21, 93–107. doi: 10.1080/15252019.2021.1944405

[ref1005] Cheong R. (1995). The virtual threat to travel and tourism. Tour. Manag. 16, 417–422. doi: 10.1016/0261-5177(95)00049-T

[ref17] Cheong K. Yu W. J. Sekhar S. C. Tham K. W. Kosonen R. (2007). Local thermal sensation and comfort study in a field environment chamber served by displacement ventilation system in the tropics. Build. Environ. 42, 525–533. doi: 10.1016/j.buildenv.2005.09.008

[ref18] Chinazzo G. Chamilothori K. Wienold J. Andersen M. (2020a). Temperature–color interaction: subjective indoor environmental perception and physiological responses in virtual reality. Hum. Factors 63, 474–502. doi: 10.1177/0018720819892383, PMID: 31928417

[ref19] Chinazzo G. Wienold J. Andersen M. (2018). Combined effects of daylight transmitted through coloured glazing and indoor temperature on thermal responses and overall comfort. Build. Environ. 144, 583–597. doi: 10.1016/j.buildenv.2018.08.045, PMID: 31928417

[ref20] Chinazzo G. Wienold J. Andersen M. (2020b). Effect of indoor temperature and glazing with saturated color on visual perception of daylight. LEUKOS 17, 183–204. doi: 10.1080/15502724.2020.1726182

[ref21] Cho Y. H. Wang Y. Fesenmaier D. R. (2002). Searching for experiences: the web-based virtual tour in tourism marketing. J. Travel Tour. Mark. 12, 1–17. doi: 10.1300/J073v12n04_01

[ref22] Choi H. S. Kim S. H. (2016). A content service deployment plan for metaverse museum exhibitions—centering on the combination of beacons and hmds. Int. J. Inf. Manag. 37, 1519–1527. doi: 10.1016/j.ijinfomgt.2016.04.017

[ref23] Collins C. (2008). Looking to the future: higher education in the metaverse. Educ. Rev. 37, 905–917. doi: 10.1016/S0278-6915(99)00071-X

[ref24] Dade P. (2010). A dictionary of psychology. Ref. Rev. 24, 20–21. doi: 10.1108/09504121011011824

[ref25] Deller S. (2010). Rural poverty, tourism and spatial heterogeneity. Ann. Tour. Res. 37, 180–205. doi: 10.1016/j.annals.2009.09.001

[ref26] Dieck D. T. Dieck M. C. T. Jung T. Moorhouse N. (2018). Tourists' virtual reality adoption: an exploratory study from Lake District National Park. Leis. Stud. 37, 371–383. doi: 10.1080/02614367.2018.1466905

[ref27] Dieck M. C. T. Jung T. (2018). A theoretical model of mobile augmented reality acceptance in urban heritage tourism. Curr. Issue Tour. 21, 154–174. doi: 10.1080/13683500.2015.1070801

[ref28] Dinh H. Q. Walker N. Hodges L. F. Song C. Kobayashi A. (1999). “Evaluating the importance of multi-sensory input on memory and the sense of presence in virtual environments.” in *Proceedings IEEE Virtual Reality*. 222–228.

[ref29] Dionisio J. D. N. William G. B. Gilbert R. (2013). 3D virtual worlds and the metaverse: current status and future possibilities. ACM Comput. Surv. 45, 1–38. doi: 10.1145/2480741.2480751

[ref30] Dueholm J. Smed K. M. (2014). Heritage authenticities–a case study of authenticity perceptions at a Danish heritage site. J. Herit. Tour. 9, 285–298. doi: 10.1080/1743873X.2014.905582

[ref31] El-Said O. Aziz H. (2021). Virtual tours a means to an end: an analysis of virtual tours' role in tourism recovery post covid-19. J. Travel Res. 61, 528–548. doi: 10.1177/0047287521997567

[ref32] Fabbri K. Ugolini A. Iacovella A. Bianchi A. P. (2020). The effect of vegetation in outdoor thermal comfort in archaeological area in urban context. Build. Environ. 175:106816. doi: 10.1016/j.buildenv.2020.106816

[ref33] Fanger P. O. (1967). Calculation of thermal comfort: introduction of a basic comfort equation. ASHRAE Trans. 73, 1–4.

[ref34] Fanger P. O. Breum N. O. Jerking E. (1977). Can colour and noise influence man's thermal comfort? Ergonomics 20, 11–18. doi: 10.1080/00140137708931596, PMID: 837904

[ref35] Fei T. He Z. Liu A. Nee A. (2019). Digital twin in industry: state-of-the-art. IEEE Trans. Indus. Inform. 15, 2405–2415. doi: 10.1109/TII.2018.2873186

[ref36] Field A. (2009). Discovering Statistics Using SPSS. London, UK: SAGE

[ref37] Frontczak M. Wargocki P. (2011). Literature survey on how different factors influence human comfort in indoor environments. Build. Environ. 46, 922–937. doi: 10.1016/j.buildenv.2010.10.021, PMID: 27409075

[ref38] Gagge A. P. Stolwijk J. Saltin B. (1969). Comfort and thermal sensations and associated physiological responses during exercise at various ambient temperatures. Environ. Res. 2, 209–229. doi: 10.1016/0013-9351(69)90037-1, PMID: 5788908

[ref1004] Galagoda R. U. Jayasinghe G. Y. Halwatura R. U. Rupasinghe H. T. (2018). The impact of urban green infrastructure as a sustainable approach towards tropical micro-climatic changes and human thermal comfort. Urban For. Urban Green. 34, 1–9. doi: 10.1016/j.ufug.2018.05.008

[ref39] Gao H. J. Li J. Y. (2017). The correlation between tourists′ emotion and climate comfort index based on the micro-blog big data:a case study of domestic tourists in xi′an city. J. Shaanxi Norm. Univ. 45, 110–117. doi: 10.15983/j.cnki.jsnu.2017.01.414

[ref40] Gao Z. Wang H. Lv H. Wang M. Qi Y. (2022). Evaluating the effects of non-isomorphic rotation on 3d manipulation tasks in mixed reality simulation. IEEE Trans. Vis. Comput. Graph. 28, 1261–1273. doi: 10.1109/TVCG.2020.3010247, PMID: 32746279

[ref41] Girvan C. (2018). What is a virtual world? Definition and classification. Educ. Technol. Res. Dev. 66, 1087–1100. doi: 10.1007/s11423-018-9577-y, PMID: 35629984

[ref42] González-Rodríguez M. R. Díaz-Fernández M. C. Pino-Mejías M. Á. (2020). The impact of virtual reality technology on tourists’ experience: a textual data analysis. Soft. Comput. 24, 13879–13892. doi: 10.1007/s00500-020-04883-y

[ref1003] Grimm A. M. Zilli M. T. (2009). Interannual variability and seasonal evolution of summer monsoon rainfall in South America. J. Clim. 22, 2257–2275. doi: 10.1175/2008JCLI2345.1

[ref43] Gssling S. Scott D. Hall C. M. (2020). Pandemics, tourism and global change: a rapid assessment of COVID-19. J. Sustain. Tour. 29, 1–20. doi: 10.1080/09669582.2020.1758708

[ref44] Guan H. Y. Hu S. T. Lu M. L. He M. Y. Mao Z. Liu G. D. (2020). People's subjective and physiological responses to the combined thermal-acoustic environments. Build. Environ. 172:106709. doi: 10.1016/j.buildenv.2020.106709

[ref45] Gutierrez-Avellanosa D. H. Bennadji A. (2015). Analysis of indoor climate and occupants' behaviour in traditional Scottish dwellings. Energy Procedia 78, 639–644. doi: 10.1016/j.egypro.2015.11.046

[ref46] Guttentag D. A. (2010). Virtual reality: applications and implications for tourism. Tour. Manag. 31, 637–651. doi: 10.1016/j.tourman.2009.07.003, PMID: 35844881

[ref47] Hemmatjo R. Motamedzade M. Aliabadi M. Kalatpour O. Farhadian M. (2017). The effect of artificial smoke compound on physiological responses, cognitive functions and work performance during firefighting activities in a smoke-diving room: an intervention study. Int. J. Occup. Saf. Ergon. 24, 358–365. doi: 10.1080/10803548.2017.1299995, PMID: 28278005

[ref48] Hendler J. (2009). Web 3.0 Emerging. Computer 42, 111–113. doi: 10.1109/MC.2009.30, PMID: 36448315

[ref49] Houghten F. C. Yaglou C. P. (1923). Determining lines of equal comfort. ASHVE Transac. 29, 163–176.

[ref50] Huang Y. C. Backman S. J. Backman K. F. Moore D. (2013). Exploring user acceptance of 3D virtual worlds in travel and tourism marketing. Tour. Manag. 36, 490–501. doi: 10.1016/j.tourman.2012.09.009

[ref51] Huang X. T. Wei Z. D. Leung X. Y. (2020). What you feel may not be what you experience: a psychophysiological study on flow in VR travel experiences. Asia Pac. J. Tour. Res. 25, 736–747. doi: 10.1080/10941665.2019.1711141, PMID: 36455018

[ref52] Jansson A. (2002). Spatial phantasmagoria - the mediatization of tourism experience. Eur. J. Commun. 17, 429–443. doi: 10.1177/02673231020170040201

[ref53] Jendritzky G. De D. R. Havenith G. (2012). Utc—why another thermal index? Int. J. Biometeorol. 56, 421–428. doi: 10.1007/s00484-011-0513-7, PMID: 22187087

[ref54] Ji L. D. Wu D. D. Xie H. B. Yao B. B. Chen Y. M. Irwin D. M. (2020). Ambient temperature is a strong selective factor influencing human development and immunity. Genom. Proteom. Bioinform. 18, 489–500. doi: 10.1016/j.gpb.2019.11.009, PMID: 32822870 PMC8377383

[ref55] Joo-Eon J. (2021). The effects of user experience-based design innovativeness on user–Metaverse Platform Channel relationships in South Korea. J. Distribut. Sci. 19, 81–90. doi: 10.15722/jds.19.11.202111.81

[ref56] Kilchenmann L. Senn O. (2015). Microtiming in swing and funk affects the body movement behavior of music expert listeners. Front. Psychol. 6:1232. doi: 10.3389/fpsyg.2015.01232, PMID: 26347694 PMC4542135

[ref57] Kim J. Hong T. Kong M. Jeong K. (2020). Building occupants' psycho-physiological response to indoor climate and CO2 concentration changes in office buildings. Build. Environ. 169:106596. doi: 10.1016/j.buildenv.2019.106596

[ref58] Kim M. J. Lee C. K. Jung T. (2020). Exploring consumer behavior in virtual reality tourism using an extended stimulus-organism-response model. J. Travel Res. 59, 69–89. doi: 10.1177/0047287518818915

[ref59] Lam C. Gallant A. Tapper N. J. (2018). Perceptions of thermal comfort in heatwave and non-heatwave conditions in Melbourne, Australia. Urban Clim. 23, 204–218. doi: 10.1016/j.uclim.2016.08.006

[ref60] Lee H. Jung T. H. Dieck M. Chung N. (2020). Experiencing immersive virtual reality in museums. Inf. Manag. 57:103229. doi: 10.1016/j.im.2019.103229, PMID: 36277032

[ref61] Lin L. P. Huang S. C. Ho Y. C. (2020). Could virtual reality effectively market slow travel in a heritage destination? Tour. Manag. 78:104027. doi: 10.1016/j.tourman.2019.104027

[ref62] Liu Y. P. Hu M. J. Zhao B. (2020). Interactions between forest landscape elements and eye movement behavior under audio-visual integrated conditions. J. For. Res. 25, 21–30. doi: 10.1080/13416979.2019.1707341

[ref63] Loureiro S. Guerreiro J. Ali F. (2020). 20 years of research on virtual reality and augmented reality in tourism context: a text-mining approach. Tour. Manag. 77:104028. doi: 10.1016/j.tourman.2019.104028

[ref64] Lu S. L. Xia H. W. Wei S. S. Fang K. Qi Y. F. (2016). Analysis of the differences in thermal comfort between locals and tourists and genders in semi-open spaces under natural ventilation on a tropical island. Energ. Buildings 129, 264–273. doi: 10.1016/j.enbuild.2016.08.002

[ref65] Ma X. Fukuda H. Zhou D. Wang M. (2019). Study on outdoor thermal comfort of the commercial pedestrian block in hot-summer and cold-winter region of southern China-a case study of the Taizhou old block. Tour. Manag. 75, 186–205. doi: 10.1016/j.tourman.2019.05.005

[ref66] Marasco A. Buonincontri P. van Niekerk M. Orlowski M. Okumus F. (2018). Exploring the role of next-generation virtual technologies in destination marketing. J. Destin. Mark. Manag. 9, 138–148. doi: 10.1016/j.jdmm.2017.12.002

[ref67] Martinez-Grana A. M. Goy J. L. Cimarra C. A. (2013). A virtual tour of geological heritage: Valourising geodiversity using Google earth and QR code. Comput. Geosci. 61, 83–93. doi: 10.1016/j.cageo.2013.07.020

[ref1002] Matsubara M. Oguchi M. (2010). Evaluation of metaverse server in a widely-distributed environment. Springer-Verlag. 6428, 307–316. doi: 10.1007/978-3-642-16961-8_49

[ref68] Mieczkowski Z. (2010). The tourism climatic index: a method of evaluating world climates for tourism. Can. Geogr. 29, 220–233. doi: 10.1111/j.1541-0064.1985.tb00365.x

[ref69] Mine M. Rose D. Bei Y. Vanbaar J. Grundhfer A. (2012). Projection-based augmented reality in disney theme parks. Computer 45, 32–40. doi: 10.1109/MC.2012.154

[ref70] Mueller M. (1998). Media technology and society: a history: from the telegraph to the internet. Isis 90, 793–794. doi: 10.1086/384526

[ref71] Nikolopoulou M. Steemers K. (2003). Thermal comfort and psychological adaptation as a guide for designing urban spaces. Energ. Buildings 35, 95–101. doi: 10.1016/S0378-7788(02)00084-1, PMID: 32640397

[ref72] Nolin P. Stipanicic A. Henry M. Lachapelle Y. Lussier-Desrochers D. Allain P. (2016). ClinicaVR: classroom-CPT: a virtual reality tool for assessing attention and inhibition in children and adolescents. Comput. Hum. Behav. 59, 327–333. doi: 10.1016/j.chb.2016.02.023

[ref73] Oquendo-Di C. V. Olivieri F. Ruiz-Garcia L. (2022). Systematic review of the impact of green walls on urban comfort: temperature reduction and noise attenuation. Renew. Sust. Energ. Rev. 162:112463. doi: 10.1016/j.rser.2022.112463

[ref74] Peng L. Weng J. Yang Y. Wen H. W. (2022). Impact of light environment on Driver's physiology and psychology in interior zone of long tunnel. Front. Public Health 10:842750. doi: 10.3389/fpubh.2022.842750, PMID: 35309214 PMC8927641

[ref75] Perch-Nielsen S. L. Amelung B. Knutti R. (2010). Future climate resources for tourism in Europe based on the daily tourism climatic index. Clim. Chang. 103, 363–381. doi: 10.1007/s10584-009-9772-2

[ref76] Perkins D. R. Debbage K. G. (2016). Weather and tourism: thermal comfort and zoological park visitor attendance. Atmosphere 7:44. doi: 10.3390/atmos7030044

[ref77] Perry H. J. S. Williams A. P. (1995). Virtual reality: a new horizon for the tourism industry[J]. J. Vacat. Mark. 1, 124–135. doi: 10.1177/135676679500100202

[ref78] Qu X. (2010). Low-level noise affects balance control differently when applied at different body parts. J. Biomech. 43, 2936–2940. doi: 10.1016/j.jbiomech.2010.07.010, PMID: 20705293

[ref79] Ravicz M. E. Cheng J. T. Rosowski J. J. (2014). Sound pressure distribution within natural and artificial human ear canals: forward stimulation. J. Acoust. Soc. Am. 136, 3132–3146. doi: 10.1121/1.4898420, PMID: 25480061 PMC4257973

[ref80] Razmerita L. Kirchner K. Sudzina F. (2009). Personal knowledge management the role of web 2.0 tools for managing knowledge at individual and organisational levels. Online Inf. Rev. 33, 1021–1039. doi: 10.1108/14684520911010981, PMID: 36203665

[ref81] Ren C. Z. O'Neill M. S. Park S. K. Sparrow D. Vokonas P. Schwartz J. (2011). Ambient temperature, air pollution, and heart rate variability in an aging population. Am. J. Epidemiol. 173, 1013–1021. doi: 10.1093/aje/kwq477, PMID: 21385834 PMC3121221

[ref82] Richter K. F. Winter S. Santosa S. (2011). Hierarchical representations of indoor spaces. Environ. Plan. B Plan. Design 38, 1052–1070. doi: 10.1068/b37057, PMID: 33688299

[ref83] Ritchie J. Tung V. Ritchie R. (2011). Tourism experience management research: emergence, evolution and future directions. Int. J. Contemp. Hosp. Manag. 23, 419–438. doi: 10.1108/09596111111129968

[ref84] Riva G. Gaggioli A. Grassi A. Raspelli S. Donvito G. (2011). NeuroVR 2-a free virtual reality platform for the assessment and treatment in behavioral health care. Stud. Health Technol. Inform. 163, 493–495. doi: 10.3233/978-1-60750-706-2-493, PMID: 21335845

[ref85] Riva G. Mantovani F. Gaggioli A. (2004). Presence and rehabilitation: toward second-generation virtual reality applications in neuropsychology. J. Neuroeng. Rehabil. 1:9. doi: 10.1186/1743-0003-1-9, PMID: 15679950 PMC546411

[ref86] Russell S. (2017). Artificial intelligence: the future is superintelligent. Nature 548, 520–521. doi: 10.1038/548520a

[ref87] Saposnik G. Mcilroy W. E. Teasell R. Thorpe K. E. Bayley M. Cheung D. (2010). Effectiveness of virtual reality using wii gaming technology in stroke rehabilitation: a pilot randomized clinical trial and proof of principle. Stroke 41, 1477–1484. doi: 10.1161/STROKEAHA.110.584979, PMID: 20508185 PMC4879973

[ref88] Schott C. Marshall S. (2018). Virtual reality and situated experiential education: a conceptualization and exploratory trial. J. Comp. Assist. Lrarn. 34, 843–852. doi: 10.1111/jcal.12293

[ref89] Schwarz A. Schwarz C. Jung Y. Pérez B. Wiley-Patton S. (2012). Towards an understanding of assimilation in virtual worlds: the 3c approach. Eur. J. Inf. Syst. 21, 303–320. doi: 10.1057/ejis.2011.49

[ref90] Scott D. Lemieux C. (2010). Weather and climate information for tourism. Procedia Environ. Sci. 1, 146–183. doi: 10.1016/j.proenv.2010.09.011

[ref91] Shi C. Y. Zhang J. Shen Z. P. Zhong J. (2005). Review of the studies on the tourism spatial competition and cooperation. Geogr. Geo-Info. Sci. 15, 71–79. doi: 10.1007/BF02873109

[ref92] Sigala M. (2020). Tourism and COVID-19: impacts and implications for advancing and resetting industry and research. J. Bus. Res. 117, 312–321. doi: 10.1016/j.jbusres.2020.06.015, PMID: 32546875 PMC7290228

[ref93] Singh R. Javaid M. Kataria R. Tyagi M. Haleem A. Suman R. (2020). Significant applications of virtual reality for COVID-19 pandemic. Diabetes Metab. Syndr. Clin. Res. Rev. 14, 661–664. doi: 10.1016/j.dsx.2020.05.011, PMID: 32438329 PMC7214336

[ref94] Soica S. (2016). Tourism as practice of making meaning. Ann. Tour. Res. Soc. Sci. J. 61, 96–110. doi: 10.1016/j.annals.2016.09.003, PMID: 35910042

[ref95] Steuer J. (1992). Defining virtual reality: dimensions determining telepresence. J. Commun. 42, 73–93. doi: 10.1111/j.1460-2466.1992.tb00812.x

[ref96] Sun C. Lian Z. (2016). Sensitive physiological indicators for human visual comfort evaluation. Light. Res. Technol. 48, 726–741. doi: 10.1177/1477153515624266

[ref97] Suzuki S. N. Kanematsu H. Barry D. M. Ogawa N. Yoshitake M. (2020). Virtual experiments in metaverse and their applications to collaborative projects: the framework and its significance. Proc. Comp. Sci. 176, 2125–2132. doi: 10.1016/j.procs.2020.09.249

[ref98] Takakura J. Nishimura T. Choi D. Egashira Y. Watanuki S. (2015). Nonthermal sensory input and altered human thermoregulation: effects of visual information depicting hot or cold environments. Int. J. Biometeorol. 59, 1453–1460. doi: 10.1007/s00484-015-0956-3, PMID: 25609478

[ref99] Tan Y. L. Jia J. Y. Peng S. Huang A. M. Li G. Y. (2014). Survey on some key technologies of virtual tourism system based on Web3D. J. Syst. Simulat. 26, 1541–1548. doi: 10.16182/j.cnki.joss.2014.07.030

[ref1001] Tavakoli R. Mura P. (2015). ‘Journeys in second life’-Iranian Muslim women’s behaviour in virtual tourist destinations. Tour. Manag. 46, 398–407. doi: 10.1016/j.tourman.2014.07.015

[ref100] Tetsuo K. Ryuhei T. Koichi I. Hajime H. Yasuyuki K. (1998). Estimation of thermal sensation during varied air temperature conditions. Appl. Hum. Sci. J. Physiol. Anthropol. 17, 73–78. doi: 10.2114/jpa.17.73, PMID: 9611371

[ref101] Trunfio M. Campana S. (2020). A visitors’experience model for mixed reality in the museum. Curr. Issue Tour. 23, 1053–1058. doi: 10.1080/13683500.2019.1586847

[ref102] Tsutsumi J. Nakamatsu R. Arakawa R. (2005). Thermal comfort sensations of tourists in a subtropical region. Elsevier Ergonom. Book 3, 217–224. doi: 10.1016/S1572-347X(05)80036-9

[ref103] Tsutsumi H. Tanabe S. I. Harigaya J. Iguchi Y. Nakamura G. (2007). Effect of humidity on human comfort and productivity after step changes from warm and humid environment. Build. Environ. 42, 4034–4042. doi: 10.1016/j.buildenv.2006.06.037

[ref104] Tussyadiah I. P. Dan W. Jung T. H. Dieck M. (2018). Virtual reality, presence, and attitude change: empirical evidence from tourism. Tour. Manag. 66, 140–154. doi: 10.1016/j.tourman.2017.12.003

[ref105] Vera A. H. Simon H. A. (1993). Situated action: a symbolic interpretation. Cogn. Sci. 17, 7–48. doi: 10.1207/s15516709cog1701_2

[ref106] Wagler A. Hanus M. D. (2018). Comparing virtual reality tourism to real-life experience: effects of presence and engagement on attitude and enjoyment. Commun. Res. Rep. 35, 456–464. doi: 10.1080/08824096.2018.1525350

[ref107] Walther E. Goestchel Q. (2018). The pet comfort index: questioning the model. Build. Environ. 137, 1–10. doi: 10.1016/j.buildenv.2018.03.054

[ref108] Wang H. Liu G. Hu S. Liu C. (2018). Experimental investigation about thermal effect of colour on thermal sensation and comfort. Energ. Buildings 173, 710–718. doi: 10.1016/j.enbuild.2018.06.008

[ref109] Wang L. Wang J. Huang X. Chi H. (2022). Environmental temperature in thermal comfort under different virtual tourism activity intensities:based on microclimate simulation experiment. Front. Neurosci. 15:762322. doi: 10.3389/fnins.2021.762322, PMID: 35173571 PMC8841518

[ref110] Wang B. Zacharias J. (2020). Noise, odor and passenger density in perceived crowding in public transport. Transp. Res. A Policy Pract. 135, 215–223. doi: 10.1016/j.tra.2020.03.013

[ref111] Williams P. Hobson J. P. (1995). Virtual reality and tourism: fact or fantasy? Tour. Manag. 16, 423–427. doi: 10.1016/0261-5177(95)00050-X, PMID: 36436469

[ref112] Xiong J. Lian Z. Zhou X. You J. Lin Y. (2016). Potential indicators for the effect of temperature steps on human health and thermal comfort. Energ. Buildings 113, 87–98. doi: 10.1016/j.enbuild.2015.12.031

[ref113] Xu Q. Chen L. Chen H. Dewancker B. J. (2021). Exercise thermal sensation: physiological response to dynamic–static steps at moderate exercise. Int. J. Environ. Res. Public Health 18:4239. doi: 10.3390/ijerph18084239, PMID: 33923594 PMC8073928

[ref114] Yang Y. L. Hu L. Zhang R. Zhu X. L. Wang M. Y. (2016). Investigation of students' short-term memory performance and thermal sensation with heart rate variability under different environments in summer. Build. Environ. 195:107765. doi: 10.1016/j.buildenv.2021.107765

[ref115] Yang W. Kang J. (2005). Acoustic comfort evaluation in urban open public spaces. Appl. Acoust. 66, 211–229. doi: 10.1016/j.apacoust.2004.07.011

[ref116] Yang M. Kang J. (2013). Psychoacoustical evaluation of natural and urban sounds in soundscapes. J. Acoust. Soc. Am. 134, 840–851. doi: 10.1121/1.4807800, PMID: 23862890

[ref117] Yang X. Z. Lin L. U. Zhang G. S. Song L. U. Xuan G. F. (2004). Study on spatial structure of tourism resources in Zhoushan archipelago. Geogr. Geo-Info. Sci. 20:87. doi: 10.2174/0929866043478455

[ref118] Yang W. Moon H. J. (2019). Effects of recorded water sounds on intrusive traffic noise perception under three indoor temperatures. Appl. Acoust. 145, 234–244. doi: 10.1016/j.apacoust.2018.10.015

[ref119] Yu D. D. Li S. (2019). Scale of human thermal sensation using seasonal anchor method: a chinese case study. J. Nat. Resour. 34, 1633–1653. doi: 10.31497/zrzyxb.20190806

[ref120] Yun G. Y. Kong H. J. Kim J. T. (2012). The effect of seasons and prevailing environments on adaptive comfort temperatures in open plan offices. Indoor Built Environ. 21, 41–47. doi: 10.1177/1420326X11419929

[ref121] Zhang H. Huizenga C. Arens E. Wang D. (2004). Thermal sensation and comfort in transient non-uniform thermal environments. Eur. J. Appl. Physiol. 92, 728–733. doi: 10.1007/s00421-004-1137-y, PMID: 15221406

[ref122] Zhang Y. F. Zhao R. (2008). Overall thermal sensation, acceptability and comfort. Build. Environ. 43, 44–50. doi: 10.1016/j.buildenv.2006.11.036, PMID: 36438077

[ref123] Zheng P. Ma Y. F. Li T. S. (2010). Virtual becoming reality:thoughts about the study kernel and category of virtual tourism. Tour. Trib. 25, 13–18. doi: 10.3969/j.issn.1002-5006.2010.02.007

[ref124] Zhong L. S. Yu H. Zeng Y. X. (2019). Impact of climate change osn Tibet tourism based on tourism climate index. J. Geogr. Sci. 29, 2085–2100. doi: 10.1007/s11442-019-1706-y

[ref125] Zyda M. (2005). From visual simulation to virtual reality to games. Computer 38, 25–32. doi: 10.1109/MC.2005.297, PMID: 36304263

